# Quantitative High-Throughput Identification of Drugs as Modulators of Human Constitutive Androstane Receptor

**DOI:** 10.1038/srep10405

**Published:** 2015-05-20

**Authors:** Caitlin Lynch, Jinghua Zhao, Ruili Huang, Jingwei Xiao, Linhao Li, Scott Heyward, Menghang Xia, Hongbing Wang

**Affiliations:** 1 Department of Pharmaceutical Sciences, University of Maryland School of Pharmacy, Baltimore, 21201 Maryland; 2 National Center for Advancing Translational Sciences (NCATS), National Institutes of Health, Bethesda, 20892 Maryland; 3 Bioreclamation IVT, Baltimore, 20 Maryland

## Abstract

The constitutive androstane receptor (CAR, NR1I3) plays a key role in governing the transcription of numerous hepatic genes that involve xenobiotic metabolism/clearance, energy homeostasis, and cell proliferation. Thus, identification of novel human CAR (hCAR) modulators may not only enhance early prediction of drug-drug interactions but also offer potentially novel therapeutics for diseases such as metabolic disorders and cancer. In this study, we have generated a double stable cell line expressing both hCAR and a CYP2B6-driven luciferase reporter for quantitative high-throughput screening (qHTS) of hCAR modulators. Approximately 2800 compounds from the NIH Chemical Genomics Center Pharmaceutical Collection were screened employing both the activation and deactivation modes of the qHTS. Activators (115) and deactivators (152) of hCAR were identified from the primary qHTS, among which 10 agonists and 10 antagonists were further validated in the physiologically relevant human primary hepatocytes for compound-mediated hCAR nuclear translocation and target gene expression. Collectively, our results reveal that hCAR modulators can be efficiently identified through this newly established qHTS assay. Profiling drug collections for hCAR activity would facilitate the prediction of metabolism-based drug-drug interactions, and may lead to the identification of potential novel therapeutics.

The constitutive androstane receptor (CAR, NR1I3) is well-recognized as a xenobiotic receptor that coordinates comprehensive metabolic responses in the liver when exposed to exogenous compounds including clinically used drugs and environmental chemicals^1^,^2^,^3^. Upon activation, CAR regulates the transcription of genes encoding drug metabolizing enzymes such as cytochrome P450s (CYP) and uridine diphosphate glucuronosyltransferases, as well as drug transporters such as multidrug resistance-associated proteins by binding to specific response elements located in their respective promoter regions^4^,^5^,^6^. Compounds which activate CAR may accelerate the metabolism and elimination of co-administered drugs and cause unexpected drug-drug interactions (DDI) leading to decreased therapeutic efficacy or enhanced toxicity^7^. Accumulating evidence reveals that CAR has evolved into a modulator dictating both xenobiotic and endobiotic stimulations by regulating the transcription of genes associated with drug uptake, metabolism, and excretion, as well as energy homeostasis, cell proliferation and tumor development^8^,^9^,^10^,^11^. Thus, identification of small molecules as CAR activators or deactivators is beneficial for early prediction of metabolism-based DDI and for the development of CAR modulators as potential drug candidates.

Although the endobiotic function of CAR is rather solidly established in rodent animal models, significant species-specific differences between human CAR (hCAR) and its rodent counterparts hinder the extrapolation of such findings from mouse to human. For instance, 1,4-bis(2-(3,5-dichloropyridyloxy))benzene (TCPOBOP) and estradiol activate mouse but not human CAR, while androstanol and progesterone repress the activity of mouse but not human CAR at pharmacological concentrations^12^,^13^. On the other hand, 6-(4-chlorophenyl) imidazo[2,1-b][1,3]-thiazole-5-carbaldehyde-O-(3,4-dichlorobenzyl)oxime (CITCO), a selective hCAR agonist, has no influence on the activity of mouse CAR (mCAR)^14^. In addition to the species selectivity in ligand binding and activation of CAR, human and mouse CAR also exhibit differences in target gene regulation. Activation of mCAR by TCPOBOP significantly alleviates high fat diet-induced obesity and type 2 diabetes through a coordinated repression of genes associated with lipogenesis, fatty acid synthesis, and gluconeogenesis^15^,^16^. In contrast, our recent findings demonstrate that activation of hCAR selectively inhibits gluconeogenesis without suppressing either fatty acid synthesis or lipogenesis^17^. Moreover, while TCPOBOP- and phenobarbital (PB)-induced tumor promotion in mice is mCAR dependent, activation of hCAR by CITCO is associated with cell cycle arrest and enhanced apoptosis in human brain tumor stem cells^18^ as well as in hCAR transgenic mice (data not shown). Together, these studies suggest that pronounced species variations may exist regarding the role of CAR in energy metabolism and cell proliferation.

Despite an escalating interest in the biological roles of CAR, a relatively limited number of CAR modulators has been reported thus far. This phenomenon is partially attributed to the fact that: 1) unlike classical nuclear receptors, CAR is spontaneously accumulated inside the nucleus and constitutively activated in immortalized cell lines without ligand activation^19^,^20^; 2) structurally, CAR has a relatively small ligand-binding pocket (675 Å) in comparison to its sister receptor, the pregnane X receptor (PXR, 1290-1540 Å)^21^,^22^; and 3) CAR signaling can be activated via either direct ligand-binding or ligand-independent pathways^1^,^11^. In contrast to immortalized cells, CAR is sequestered in the cytoplasm of primary hepatocytes or intact liver prior to activation^19^,^20^. It is evident now that activation of CAR is a multi-step process initiated by nuclear accumulation. Although blocking nuclear translocation of CAR is a function shared by several known mCAR deactivators such as the protein phosphatase 2A inhibitor, okadaic acid^23^, the activity of nuclear localized CAR can be repressed by antagonists such as 1-(2-chlorophenylmethylpropyl)-3-isoquinoline-carboxamide (PK11195) by disrupting CAR-coactivator interactions^24^. The beneficial versus detrimental effects of CAR are directly related to the balance between physiological activation and deactivation of this receptor. To overcome aforementioned limitations of CAR, in particular towards a quantitative high-throughput screening (qHTS) format *in vitro*, we have generated a stable cell line expressing both the full-length hCAR protein, and a luciferase signal driven by the promoter of CYP2B6, the prototypical target of hCAR^25^. Application of the potent hCAR agonist CITCO and antagonist PK11195 enables the qHTS assays to identify both hCAR activators and deactivators.

In this report, we have miniaturized the cell-based transactivation assay for hCAR into a 1536-well plate format. A total of 2816 small molecule drugs from the NIH Chemical Genomics Center Pharmaceutical Collection (NPC) were profiled for their potential in modulating CAR signaling in agonist and antagonist modes, which led to the identification of many previously unreported hCAR modulators. Selected hits were subjected to hCAR nuclear translocation and target gene expression experiments in human primary hepatocytes (HPH) for further validation.

## Results

### qHTS Performance and Identification of hCAR Agonists

**Figure 1:**
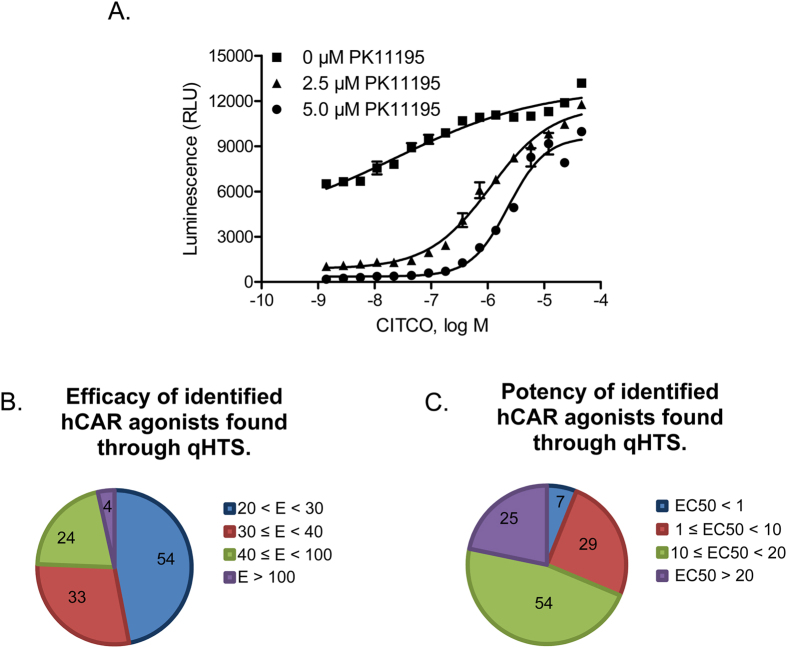
Cell-based qHTS assay optimization and hCAR agonist identification. A double stable cell line, HepG2-CYP2B6-hCAR, was generated and utilized to screen 2816 clinically used drugs for hCAR activation. Three trials of the experiment in 1536-well plate format were run using 0 μM, 2.5 μM, or 5 μM PK11195 co-treated with varying concentrations of CITCO (**A**). Luciferase activities from primary screening of the 2816 compounds were normalized and compared with the E_max_ of CITCO serving as a positive control (**B**); or categorized based on their EC_50_ values (**C**). There were 115 agonists identified and categorized based on their efficacy and potency.

Consistent with the constitutive activation of CAR in immortalized cells, the generated HepG2-CYP2B6-hCAR stable line maintains a high level of hCAR activity. In the agonist screening mode, PK11195, a known hCAR deactivator^24^, was used to lower the basal activity. As shown in Fig. 1A, PK11195 markedly reduced the hCAR activity at 2.5 μM and 5.0 μM, while co-treatment of CITCO, a potent hCAR agonist^14^, restored the hCAR activity in a concentration-dependent manner. Notably, without co-administration of PK11195, CITCO-mediated activation of hCAR was moderate due to the inherent high basal activity. In this study, 2816 drugs from the NPC library were screened in agonist mode of the qHTS assay, where 2.5 μM of PK11195 was selected to repress the basal activity of hCAR, and CITCO, which yielded an EC_50_ of 1.11 μM, was used as a positive control. The agonist screening performed well with an average signal to background (S/B) ratio of 10.0 and an average Z’ factor of 0.61. In the primary screening, 115 (4%) test compounds were identified as potential hCAR agonists based on their efficacies, in comparison to the E_max_ of CITCO, and potencies relying on their respective EC_50_ values. These compounds exhibited an efficacy at least 20% of the CITCO E_max_ and an EC_50_ ≤ 35 μM (Fig. 1B,C). Four drugs were found to have an efficacy greater than the E_max_ of CITCO, and seven with EC_50_ values less than 1 μM. These 115 potential agonists identified in the primary screening were also re-evaluated using the same qHTS method. Based on the curve class of the main readout, the agonist mode assay generated 97% reproducibility implicating the strong consistency of the screening. Ten potential agonists were selected for further evaluation based on their efficacies and potencies in the qHTS assays, pharmacological importance, and commercial availability. Table 1 depicts the potency and efficacy in hCAR activation, as well as the structural features of these 10 compounds.

### Nuclear Translocation of hCAR

**Figure 2:**
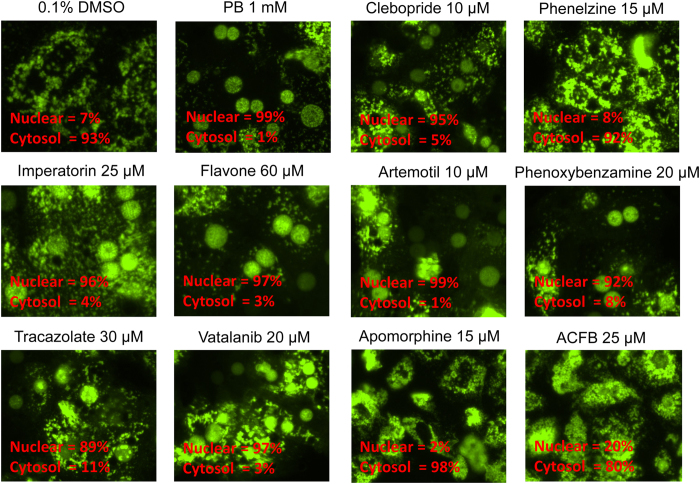
Nuclear translocation of hCAR in HPH. Cultured HPH were infected with Ad/EYFP-hCAR and treated with vehicle (0.1% DMSO), PB (1 mM) or each of the 10 selected agonists from qHTS assays at the indicated concentrations. After 24 h of treatment, hepatocytes were subjected to confocal microscopy for visualization of EYFP-hCAR localization. Approximately 100 EYFP-hCAR expressing cells from each treatment were counted according to the subcellular localization of hCAR in nucleus or cytoplasm.

Unlike the spontaneous nuclear accumulation of CAR in immortalized cells, hCAR is predominantly retained in the cytoplasm of HPH prior to activation. Chemically-stimulated nuclear translocation of hCAR in HPH has been recognized as the first step of hCAR activation. To validate the agonistic feature of compounds identified from the qHTS in a physiologically relevant cellular system, the 10 selected compounds were tested in HPH infected with the adenovirus expressing enhanced yellow fluorescent protein tagged hCAR (Ad/EYFP-hCAR)^20^. Representative images were acquired using confocal microscopy illustrating the nuclear and cytoplasmic distribution of hCAR upon chemical stimulation. As expected, EYFP-hCAR was expressed predominantly in the cytoplasm (97%) of HPH under vehicle control, and was translocated to the nucleus (99%) upon PB (a prototypical CAR activator) treatment (Fig. 2). Seven out of the ten test compounds resulted in marked accumulation of EYFP-hCAR in the nucleus to the extent (nuclear localization of 89-99%) that is comparable to that of PB. Phenelzine, apomorphine, and ACFB, which didn’t translocate hCAR, were only associated with negligible induction of hCAR target genes in HPH.

### Effects of hCAR agonists on the expression of CYP2B6 and CYP3A4

**Figure 3:**
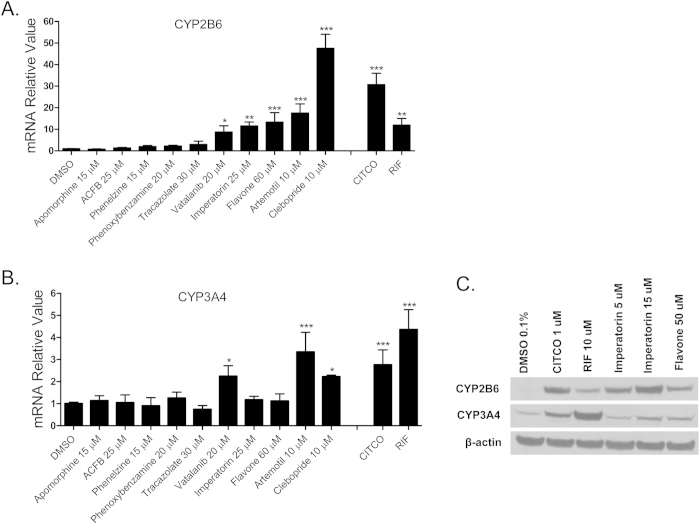
Induction of CYP2B6 and CYP3A4 expression in HPH. HPH were treated with the same 10 potential agonists selected from qHTS, CITCO (1 μM), rifampicin (10 μM), or the vehicle control (0.1% DMSO) as described in *Methods*. Real-time PCR was used to analyze the mRNA expression of CYP2B6 (A) and CYP3A4 (B), respectively. In separate experiments, induction of CYP2B6 and CYP3A4 proteins by imperatorin and flavone were detected in an immunoblotting assay (C). Each bar represents the mean±SD (n = 3). *, P < 0.05; **, P < 0.01; ***, P < 0.001.

As a promiscuous xenobiotic sensor, hCAR governs the inductive expression of many hepatic genes with CYP2B6 as the primary target^4^. Activation of hCAR also results in a moderate induction of CYP3A4^26^. To evaluate the effects of identified hCAR agonists on target gene regulation, HPH were treated with the aforementioned 10 agonists and the mRNA expression of CYP2B6 and CYP3A4 was determined. As demonstrated in Fig. 3A, five out of the ten compounds (vatalanib, imperatorin, flavone, artemotil, and clebopride) significantly induced the expression of CYP2B6, with clebopride exhibiting potent induction exceeding that of CITCO. Notably, vatalanib, artemotil, and clebopride also markedly enhanced the expression of CYP3A4 while imperatorin and flavone only exhibited marginal effect on CYP3A4 mRNA expression (Fig. 3B). The preferential induction of CYP2B6 over CYP3A4 by imperatorin and flavone was further confirmed at the protein level (Fig. 3C), implying their potential selectivity towards hCAR modulation.

### Identification of hCAR Antagonists and Viability Assays

**Figure 4:**
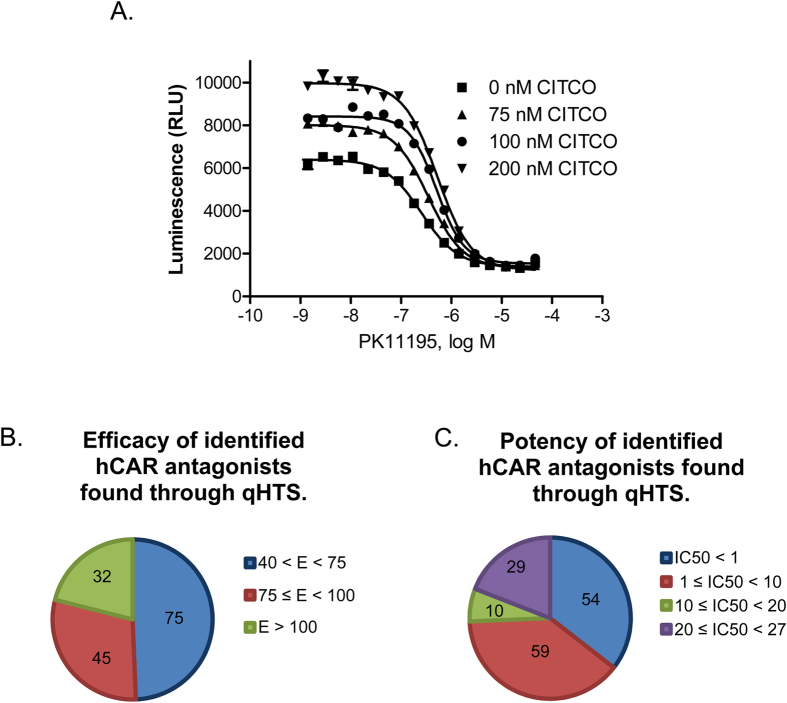
Cell-based qHTS assay optimization and hCAR antagonist identification. The double stable HepG2-CYP2B6-hCAR cell line was utilized to screen 2816 clinically used drugs in the antagonist mode. Four trials of the experiment were run using 0 μM, 0.075 μM, 0.1 μM, or 0.2 μM CITCO co-treated with varying concentrations of PK11195 for assay optimization (**A**). In the following primary screening, luciferase activities of the 2816 compounds were normalized and compared to that of PK11195 as a positive control antagonist (**B**); or classified based on their IC_50_ values (**C**). There were 152 antagonists identified and categorized based on their efficacy and potency.

The inherent high basal activity of hCAR in the HepG2-CYP2B6-hCAR cell line, though a hurdle for agonist identification, offers excellent opportunity for qHTS in antagonist mode. As shown in Fig. 4A, PK11195 concentration-dependently repressed the activity of hCAR. Co-treatment with CITCO at 75 nM, 100 nM, or 200 nM resulted in a moderate but concentration-dependent increase of the basal activity of hCAR, which extended the range of PK11195-mediated inhibition of hCAR activity. When co-treated with 100 nM of CITCO, PK11195 produced an optimal inhibition curve with an IC_50_ of 510 nM. The same 2816 drugs were screened under this experimental condition and the luciferase activity of each compound was normalized to the E_max_ of PK11195. The IC_50_ of each compound was also determined. The antagonistic qHTS assay performed well in the 1536-well plate format with S/B of 8.4, Z factor of 0.66, and a CV of 8.8%. As another measurement of assay performance, the antagonist mode assay was accompanied with a cell viability experiment to prevent artificial inhibition resulting from cytotoxicity. A set of 152 NPC compounds were identified as potential hCAR antagonists through the primary screening, of which 32 compounds had an efficacy >100%, 75 with efficacy between 75% and 100%, and 45 at the efficacy of 40% to 75% in comparison to PK11195 (Fig. 4B). Fifty-four of these compounds were found to have an IC_50_ < 1 μM and 59 had an IC_50_ between 1 and 10 μM (Fig. 4C). Similar to the agonist mode assays, the reproducibility of the assay was validated in the secondary cherry-pick assay, where the 152 antagonists were rescreened using the same assay conditions. Overall the antagonist screening generated a 98% reproducibility rate. Notably, clusters of drugs involving protease inhibition and chemotherapy were enriched in identified hCAR antagonists. Based on the combination of efficacy, potency, and cytotoxicity, 10 potential antagonists as summarized in Table 2 were selected for further validation.

### Effects of hCAR antagonists on target gene expression and PXR activation

**Figure 5:**
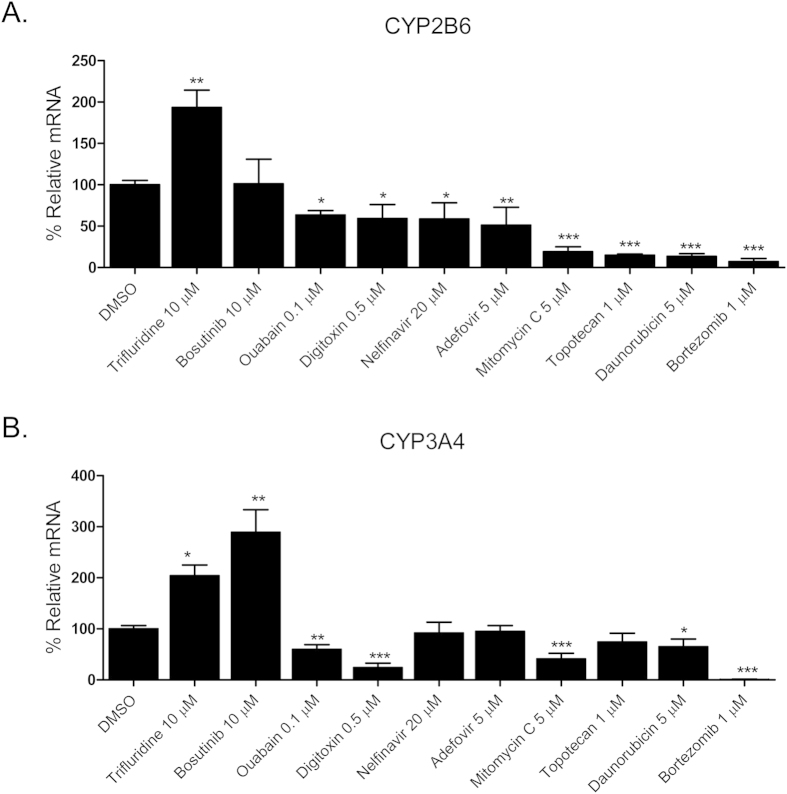
Antagonist-mediated expression of CYP2B6 and CYP3A4 in HPH. HPH were treated with the vehicle control (0.1% DMSO) or each of the 10 potential antagonists selected from the qHTS at indicated concentrations for 24 h. Real-time PCR was used to analyze the expression of CYP2B6 (**A**) and CYP3A4 (B), respectively. Data represents the mean±SD (n = 3). *, P < 0.05; **, P < 0.01; ***, P < 0.001.

**Figure 6:**
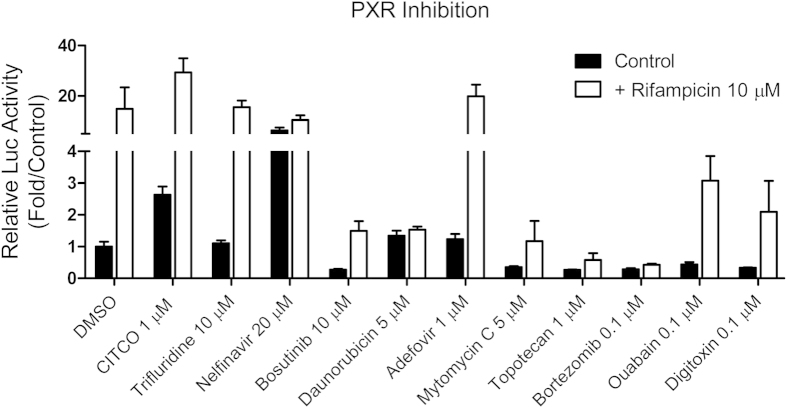
Effects of hCAR antagonists on PXR activation. HepG2 cells were transfected with hPXR expression and CYP2B6 luciferase reporter vectors as described in *Methods*. Twenty-four hours after transfection, cells were treated with vehicle control (0.1% DMSO), CITCO (1 μM), or one of the 10 antagonists at indicated concentrations for another 24 h, in the presence or absence of rifampicin (10 μM). Luciferase activities were determined and expressed relative to vehicle control. Data represents the mean±SD (n = 3).

Ten compounds from the antagonist screening were selected using criteria similar to that in the agonist mode for further evaluation. In treated HPH, eight of the 10 antagonists significantly repressed the mRNA expression of CYP2B6 while it was moderately induced by trifluridine (Fig. 5A). On the other hand, expression of CYP3A4 was differentially influenced by the same panel of compounds. The two non-inhibitors of CYP2B6 moderately induced the expression of CYP3A4, while among the eight CYP2B6 inhibitors, nelfinavir, adefovir, and topotecan had a negligible effect on mRNA expression of CYP3A4 (Fig. 5B). To confirm the repression of target gene expression was not a toxic related non-specific event, daunorubicin and bortezomib, the two most potent repressors of CYP2B6, were further subjected to MTT and gene expression analyses in HPH. Clearly, daunorubicin at 5 μM and bortezomib at 1 μM were not associated with significant cytotoxicity in HPH; marked and concentration-dependent repression of both CYP2B6 and CYP3A4 was observed (Supplementary Figure S1 and S2). To detect the potential interaction of these hCAR antagonists with PXR, a transient PXR luciferase assay was carried out. As shown in fig. 6, six of the 10 compounds inhibited both the basal and RIF-induced PXR activity, while daunorubicin only repressed RIF-induced PXR activity. Interestingly, adefovir and trifluridine had no effects on either basal or RIF-induced hPXR activation. Nelfinavir, on the other hand, induced hPXR activation to an extent closer to that of RIF.

## Discussion

Discrepancy between the heightened importance of hCAR and the lack of small molecules as known modulators is evident. In this report, we generated a double stable cell line, HepG2-CYP2B6-hCAR, and miniaturized the luciferase assay into a 1536-well plate format for qHTS of 2816 NPC compounds for their potential modulation of the hCAR signaling pathway. Under agonist and antagonist modes, full concentration-response curves in triplicate for each test compound, as well as the robust cherry-pick follow up assay, ensured the reliability and reproducibility of the screening. Select compounds identified from the primary screening were further validated in HPH cultures which express the majority of liver-enriched transcription factors and maintain physiologically relevant capacity of drug metabolism^27^,^28^. Both novel and previously reported hCAR activators and CYP2B6 inducers have been identified. Most recently, this qHTS has been approved for the Tox21 phase II program to screen a library of approximately 10,000 environmental chemicals at NCATS.

Constitutive activation in immortalized cells is a hallmark of CAR, which makes the identification of CAR activators extremely challenging. To overcome this drawback, we have previously established PK11195, one of the most commonly used ligands of peripheral benzodiazepine receptor, as a potent and selective deactivator of hCAR in different cell lines^24^. PK11195 competes directly with CITCO and disrupts the recruitment of co-activators such as steroid receptor coactivator-1 and glucocorticoid receptor interacting protein-1 to hCAR. Thus, PK11195 represents a small molecular tool which can be utilized to effectively lower the high basal hCAR activity in HepG2-CYP2B6-hCAR cells. More importantly, the repressed hCAR activity could be rescued by agonistic ligands, such as CITCO, establishing a robust agonist mode for qHTS. On the other hand, the auto-activation characteristic of hCAR in the double stable cell line serves well with the antagonist mode of screening. Our initial screening has resulted in more antagonists (152) than agonists (115) from a pool of 2816 NPC compounds. Of the 115 agonists identified, 11 are known CAR activators or CYP2B6 inducers including the antimalarial artemisinin, the antibiotics triclocarban, and the antifungal tolnaftate^29^,^30^,^31^. Notably, nocodazole, a hCAR agonist from the current study, was reported previously as a mCAR deactivator and repressed PB-induced expression of Cyp2b10^32^. These findings together indicate nocodazole may function as an agonist of hCAR but an antagonist of mCAR. Indeed, due to the significant sequence divergence of mouse and human CAR, a single compound may have opposite and species-specific effects on this receptor. For instance, meclizine, a histamine H1 receptor antagonist, was reported as an agonist for mCAR but an inverse agonist for hCAR^33^. Among the 152 antagonists, it appears that certain classes of drugs were enriched such as protease inhibitors and anticancer drugs. Given that these drugs are often associated with relatively high cytotoxicity, an accompanied assay was employed to identify potential artifacts caused by decreased cell viability. Moreover, although the number of previously reported hCAR deactivators is limited, some of these known hCAR inverse agonists or deactivators such as clotrimazole, 4-butylphenol, and phenolphthalein were recognized in the current study. Additionally, we also identified mitomycin C, a previously reported hPXR antagonist^34^, as an antagonist of hCAR, and defined it as a dual-antagonist of both receptors. Together these results reveal the ability of the hCAR qHTS in the identification of potentially novel hCAR modulators which require further validation in more physiologically relevant systems.

Unlike in immortalized cells, hCAR maintains a low basal activity and is sequestered in the cytoplasm of HPH before activation. Chemical-induced nuclear translocation of CAR in HPH has been well accepted as the essential first step of activation. Whereas no high-content imaging screening for hCAR translocation has been established yet, we have generated an adenovirus expressing EYFP-tagged hCAR that can infect HPH with high efficacy and is efficient in detecting chemical-mediated hCAR modulation^20^. Of the 10 selected hCAR agonists, seven resulted in significant nuclear accumulation of hCAR, while the other three (apomorphine, ACFB, and phenelzine) neither translocated hCAR nor induced the expression of its target genes. Given the robust metabolic capability of HPH, but not HepG2 cells, it is possible that these three drugs might be quickly converted to non-active metabolites in HPH. Examples of such compounds include the μ-opioid receptor agonists buprenorphine and diprenorphine. Though both potently activate hCAR and hPXR in HepG2 cells they fail to induce CYP2B6 and CYP3A4 genes in HPH due to rapid metabolism^35^. Cross talk between hCAR and hPXR reflects an efficient and coordinated mechanism in defending the human body from xenobiotic challenges^36^. However, this feature also generates difficulties in defining the exact biological function of these receptors; in fact there is no pure hCAR activator identified thus far. For instance, the typical hCAR agonist CITCO also activates hPXR at higher concentrations^14^, PB is a dual-activator of both hCAR and hPXR, and the known hCAR antagonists (PK11195, clotrimazole, and meclizine) exhibit potent agonistic activity towards hPXR^24^,^37^,^38^. Notably, in cross-regulation of each other’s target genes, activation of hCAR preferentially induces CYP2B6 over CYP3A4^26^. In the current study, further evaluation of ten hCAR agonists revealed that imperatorin and flavone markedly induced CYP2B6 at both mRNA and protein levels but only marginally increased the expression of CYP3A4, although both compounds moderately activated hPXR (data not shown).

In addition to direct ligand binding, hCAR signaling can be influenced by drugs via ligand-independent pathways by which protein-kinase mediated phosphorylation/dephosphorylation of the conserved threonine (Thr)-38 of hCAR governs nuclear translocation and activation of hCAR^39^. For instance, PB, the prototypical CAR activator over multiple species, doesn’t bind to CAR. More importantly, PK11195 repressed hCAR activity cannot be rescued by indirect activators such as PB^24^. Therefore, it is important to point out that the current qHTS assays identify agonists and antagonists that competitively bind to hCAR but not ligand-independent activators and deactivators. On the other hand, although the exact molecular mechanisms controlling hCAR translocation remain elusive, nuclear accumulation represents an essential step for both direct and indirect CAR modulators. It is expected that future work will integrate luciferase-based qHTS with hCAR nuclear translocation assay at the high-content screening platform to determine modulation of hCAR by compounds acting directly and indirectly on the receptor.

In summary, our data suggest that the cell-based qHTS assay is efficient in profiling a large collection of drugs to identify novel hCAR agonists and antagonists. With the increased appreciation of the biological roles of hCAR in drug metabolism/clearance, energy homeostasis, as well as cell proliferation/cancer development, this receptor has emerged as a potentially attractive drug target in addition to function as a mediator of drug toxicity and DDI. Selective and potent hCAR modulators may offer powerful research tools in our understanding of hCAR biology. However, given the unique features of hCAR in immortalized cells vs. primary hepatocytes, direct vs. indirect activations, as well as its cross talk with hPXR, we do realize both the advantage and limitation of this qHTS experiment. Future approaches utilizing assays with various endpoints are warranted to effectively identify physiologically relevant hCAR modulators.

## Methods

### Chemicals and Biological Reagents

CITCO, PK11195, phenelzine sulfate, bortezomib, daunorubicin, digitoxin, mitomycin C, ouabain octahydrate, topotecan hydrochloride, trifluridine and flavone were purchased from Sigma-Aldrich (St. Louis, MO). Adefovir dipivoxyl, bosutinib, nifuroxazide, nelfinavir mesylate and artemotil were purchased from AK Scientific (Union City, CA). Clebopride was from OChem Incorporation (Des Plaines, IL). Phenoxybenzamine hydrochloride, apomorphine hydrochloride, 2-Amino-5-chloro-2’-fluorobenzophenone (ACFB), tracazolate hydrochloride, and vatalanib dihydrochloride salt were acquired from Fisher Scientific (Pittsburgh, PA). Imperatorin came from LKT Laboratories, Inc (St. Paul, MN). G418 sulfate and blasticidin S HCl were purchased from Life Technologies (Grand Island, NY). Matrigel, insulin, and ITS+(insulin/transferrin/selenium) were bought from BD Biosciences (Bedford, MA). The dual-luciferase assay kit was obtained from Promega (Madison, WI). Antibody against CYP2B6 was purchased from Santa Cruz (Dallas, TX). The CYP3A4 antibody was obtained from Millipore Corporation (Billerica, MA), and the β-actin antibody from Sigma-Aldrich. All cell culture mediums and supplies were ordered from Life Technologies (Grand Island, NY).

### Double Stable Cell Line Generation

The pEF6/V5-hCAR expression plasmid and the pGL4.17[*luc2/Neo*]-CYP2B6-2.2kb construct containing both the PBREM and XREM^40^, were co-transfected in HepG2 cells using lipofectamine 2000 reagents (Life Technologies) following the manufacturer’s instruction. Dulbecco’s Modified Eagle Medium (DMEM) containing 10% FBS, 100 U/ml penicillin, 100 μg/ml streptomycin, G418 (0.6-1 mg/ml) and blasticidin (10 μg/ml) were used for the selection of colonies containing a mixed population of drug resistant cells. Subsequently, these cells were transferred to collagen-coated 48-well plates and continually cultured in the same media. Single colony containing both plasmids was selected and functionally verified.

### NIH Chemical Genomics Center Pharmaceutical Collection

The NIH Chemical Genomics Center Pharmaceutical Collection (NPC) was constructed in house at NIH^41^. Briefly, the NPC consists of 2816 small molecule compounds, 52% of which are drugs approved for human or animal use by the United States Food and Drug Administration (FDA), 22% are drugs approved in Europe, Canada or Japan, and the remaining 26% are drugs approved in other countries or compounds that have been tested in clinical trials. For qHTS, each compound in the NPC was prepared as two inter-plate titration series with eight 5-fold serial dilutions. The NPC plate series were stored using desiccation at room temperature for as long as 6 months when in use, or heat sealed and stored at -80 °C for long-term storage.

### CAR Luciferase Reporter Assay and qHTS

The generated stable cell line, namely HepG2-CYP2B6-hCAR, was used to identify hCAR modulators in a qHTS platform. Assays were run in both agonist and antagonist modes. In brief, HepG2-CYP2B6-hCAR cells were dispensed at 2,500 cells/well in 1,536-well plates (Greiner Bio-One North America, Monroe, NC) using a Multidrop Combi (Thermo Fisher Scientific Inc., Waltham, MA). The assay plates were incubated at 37 °C overnight before 23 nl of each compound was transferred from the compound plate to the assay plate via a pin tool station (Kalypsys, San Diego, CA), followed by the addition of PK11195 (2.5 μM) or CITCO (0.1 μM). Each NPC compound was tested at 8 concentrations ranging from 0.6 nM to 38.3 μM. The assays were incubated at 37 °C for 24 h followed by the addition of 4 μl of the ONE-Glo luciferase reagent (Promega, Madision, WI). The assay plates were incubated at room temperature for 20 min and luminescence intensity was quantified using a ViewLux plate reader (PerkinElmer, Shelton, CT).

### Cell Viability Assay

The potential cytotoxicity of the NPC compounds in HepG2-CYP2B6-hCAR cells was measured using a luciferase-coupled ATP quantitation assay (CellTiter-Glo viability assay, Promega). The change of intracellular ATP content indicates the number of metabolically competent cells. The cells were seeded at 2,500 cells/5 μl in 1536-well plates and were exposed to each test compound at concentrations and treatment duration as previously mentioned. The assay plates were incubated for 24 h at 37 °C, followed by the addition of 4 μl/well of CellTiter-Glo reagent. After 30 min incubation at RT, the luminescence intensity of the plates was measured using a ViewLux plate reader.

### qHTS Data Analysis

As described previously^42^, raw plate reads for each titration point were normalized relative to PK11195 (antagonist mode; 38 μM = 100%) or CITCO (agonist mode; 57 μM = 100%) and DMSO (0%) controls, and then corrected by applying a pattern-correction algorithm using compound-free (DMSO only) control plates placed at the beginning and end of each NPC library plate stack (final DMSO concentration of 0.46%). Concentration-response titration points for each compound were then fitted to the Hill equation, yielding half-maximal inhibition (IC_50_) or activation (EC_50_) and maximal response (efficacy) values. Compounds from the primary qHTS screen were classified into four major curve classes according to quality of curve fit, potency and efficacy. Class 1.1, 1.2, 2.1, and 2.2 curves were high-quality and thus high-confidence concentration-response curves (CRCs). The compounds in the aforementioned curve classes were considered as active, whereas compounds with class 4 curves were deemed inactive because they did not show significant activity above the noise level across the concentrations tested. All other curve classes (including those curves with single point activity) were deemed inconclusive. Active agonists and antagonists that were not apparently cytotoxic (IC_50_ viability/IC_50_ antagonist >3-fold) were selected for cherry-picking confirmation and follow up studies. Follow-up studies were performed using a new aliquot of samples to confirm sample integrity and assay reproducibility.

### Follow-up Luciferase Assays

Agonistic and antagonistic compounds identified from primary qHTS were subjected to follow-up luciferase assays to confirm the reproducibility. These compounds were prepared in 1536-well compound plates at 11-point 3-fold dilution titrations in DMSO, with final concentrations ranging from 0.6 nM to 38.3 μM. All of these compounds were retested in both HepG2-CYP2B6-hCAR and cell viability assays.

### Human Primary Hepatocyte Cultures and Treatments

Human liver tissues were obtained through the University of Maryland Pathology Biorepository and Research Shared Service with patient consent and prior approval from the Institutional Review Board at the University of Maryland, School of Medicine. HPH were isolated from human liver specimens by a modification of the two-step collagenase digestion method as described previously^43^ or obtained from Bioreclamation *In Vitro* Technologies (Baltimore, MD). Fresh HPH were seeded at 1.5 × 10^6^, 7.5 × 10^5^ or 4.7 × 10^4^ cells/well in 6-well, 12-well, or 96-well collagen coated plates, respectively. Hepatocytes were cultured for 36 h at 37 °C before treatment with specified compounds for another 24 or 72 h for detection of mRNA or protein expression in the 6-well and 12-well plates.

### Real-time PCR

Total RNA was isolated from treated hepatocytes using the TRIzol® reagent and reverse transcribed using a High Capacity cDNA Archive Kit (Applied Biosystems, Foster City, CA) following the manufacturers’ instructions. CYP2B6 and CYP3A4 mRNA expression were normalized against that of GAPDH. Real-time PCR assays were performed in 96-well optical plates on an ABI Prism 7000 Sequence Detection System with SYBR Green PCR Master Mix. Primers used for CYP2B6, CYP3A4, and GAPDH mRNA expression were described previously^35^. Induction values were calculated using the equation: Fold = 2ΔΔCt, where ΔCt represents the differences in cycle threshold numbers between CYP2B6 or CYP3A4 and GAPDH, and ΔΔCt represents the relative change in these differences between control and treatment groups.

### Nuclear Translocation of Ad/EYFP-CAR in HPH

A recombinant adenovirus expressing enhanced yellow fluorescent protein-tagged hCAR (Ad/EYFP-hCAR) was generated as described previously^20^. Hepatocytes cultured in 96-well collagen-coated plates in serum free Williams’ E medium were infected with Ad/EFYP-hCAR (1 μl/well) for 12 h before treatment with the vehicle control, DMSO (0.1%), PB (1 mM), and selected compounds for another 8 h. Subcellular localization of hCAR was visualized using a Nikon (Tokyo, Japan) C1-LU3 instrument based on an inverted Nikon Eclipse TE2000 microscope.

### Western Blot Analyses

Homogenate proteins (30 μg) from treated HPH were resolved on an SDS polyacrylamide gel and electrophoretically transferred onto Immobilon-P polyvinylidene difluoride membranes and blocked using 5% blotting-grade blocker (nonfat dry milk, Bio-RAD). Subsequently, membranes were incubated with specific antibodies against CYP2B6 (1:200), CYP3A4 (1:5000), and β-actin (1:50,000) diluted in 1% blotting-grade blocker. β-Actin was used to normalize protein loadings. Membranes were washed using 1x Tris buffered saline–Tween 20 (Bio-RAD) and incubated with horseradish peroxidase goat anti-rabbit IgG antibody diluted at 1:5000, and developed using ECL Western blotting detection reagent (Thermo-Scientific Inc).

### Transient Transfection in HepG2

HepG2 cells cultured in a 24-well plate were co-transfected with the CYP2B6-2.2 kb reporter and hPXR expression vectors using a Lipofectamine® 2000 Transfection Kit (Life Technologies) following the manufacturer’s instruction. Eighteen hours after transfection, cells were treated with vehicle control, DMSO (0.1%), RIF (10 μM), CITCO (1 μM), or the test compounds at concentrations as indicated in the figures for 24 h. Cell lysates were assayed for firefly luciferase activities normalized against the activities of co-transfected Renilla luciferase using a Dual-Luciferase Kit (Promega). Data were represented as mean±SD of three individual transfections.

### Statistical Analysis

Experimental data are presented as a mean of triplicate determinations ± S.D. unless otherwise noted. Statistical comparisons were made by one-way analysis of variance with post-hoc Dunnett’s analysis or paired t-test. The statistical significance was set at p values of <0.05 (*), <0.01 (**), or < 0.001 (***).

## Additional Information

**How to cite this article**: Lynch, C. *et al.* Quantitative High-Throughput Identification of Drugs as Modulators of Human Constitutive Androstane Receptor. *Sci. Rep.* **5**, 10405; doi: 10.1038/srep10405 (2015).

## Supplementary Material

Supplementary Information

## Figures and Tables

**Table 1:** Agonist potency (EC_50_) and efficacy (% in parenthesis) in qHTS.

**Compound Name (CAS No.)**	**Chemical Structure**	**Primary Screen Potency [μM] (Efficacy)**	**Follow-up Confirmation Potency [μM] (Efficacy)**	**Known modulator of CAR, CYP2B6, or CYP3A4?**
Apomorphine (314-19-2)	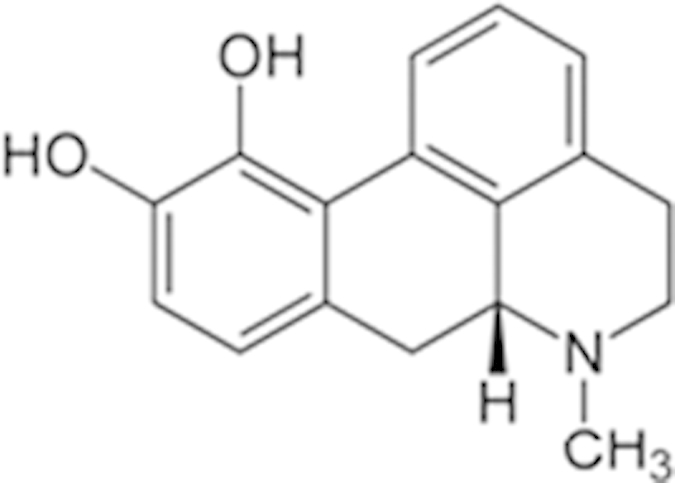	19 (21%)	13.3 (38%)	
2-Amino-5-chloro-2’-fluorobenzophenone ()	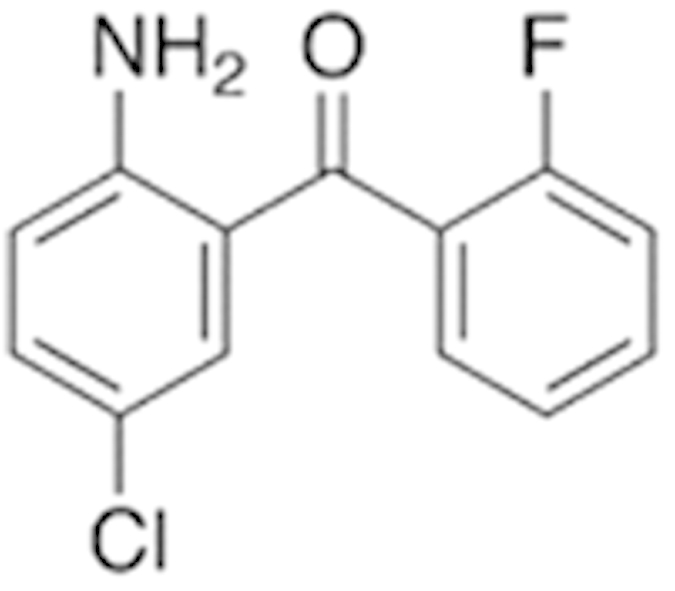	3.4 (60%)	15.6 (67%)	
Phenelzine (156-51-4)	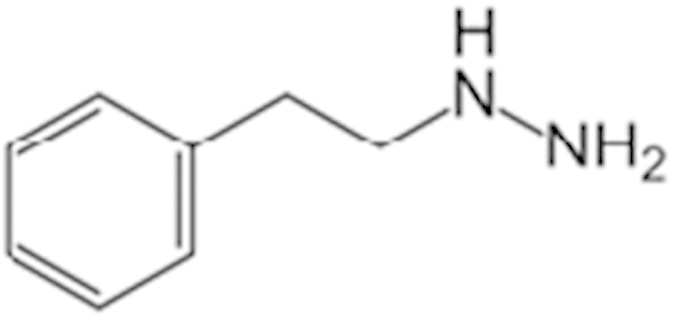	15.1 (145%)	15.9 (70%)	CYP3A4 weak inhibitor
Phenoxybenzamine (63-92-3)	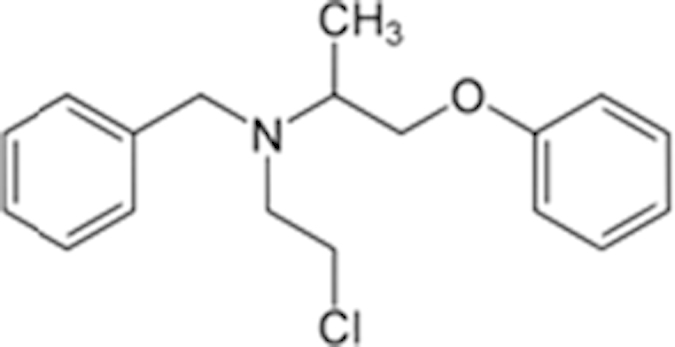	9.5 (57%)	12 (49%)	
Tracazolate (41094-88-6)	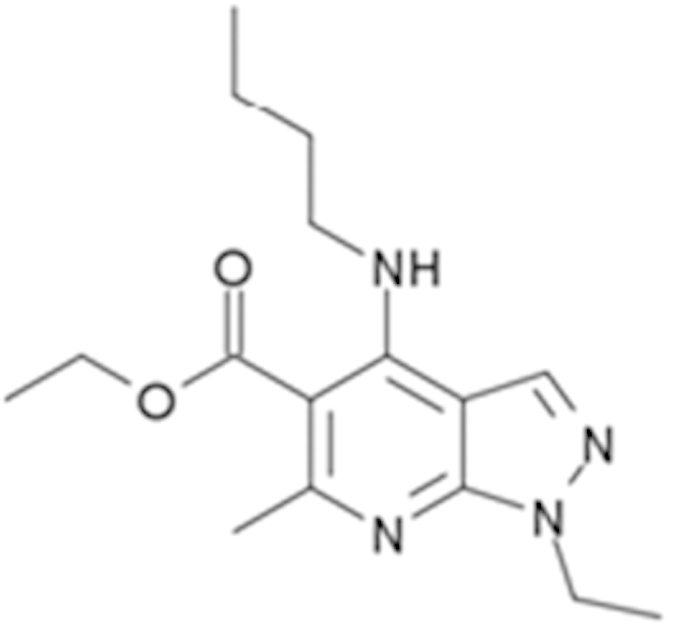	17 (27%)	24 (101%)	
Vatalanib (212141-51-0)	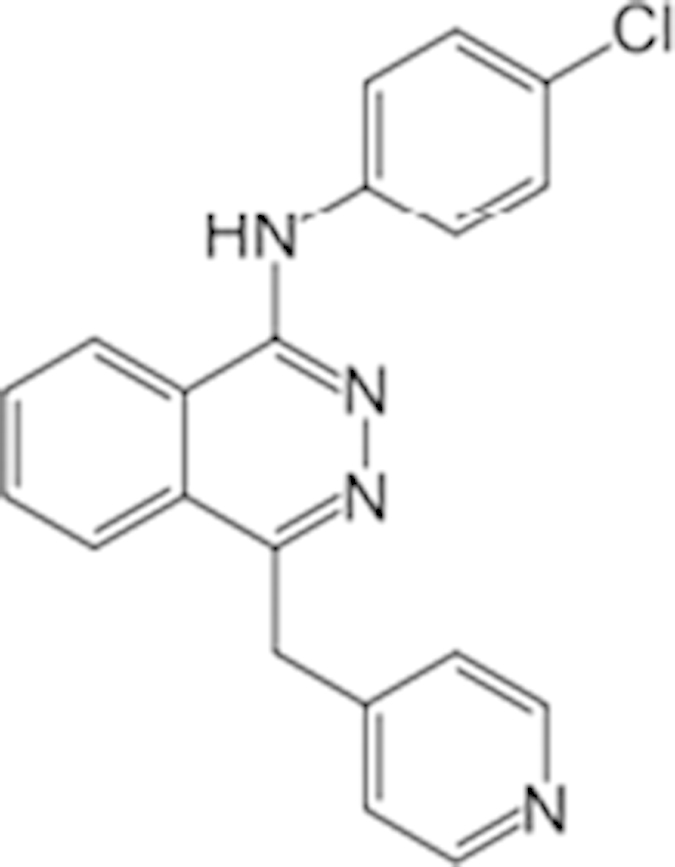	10.7 (68%)	12.6 (97%)	
Imperatorin (482-44-0)	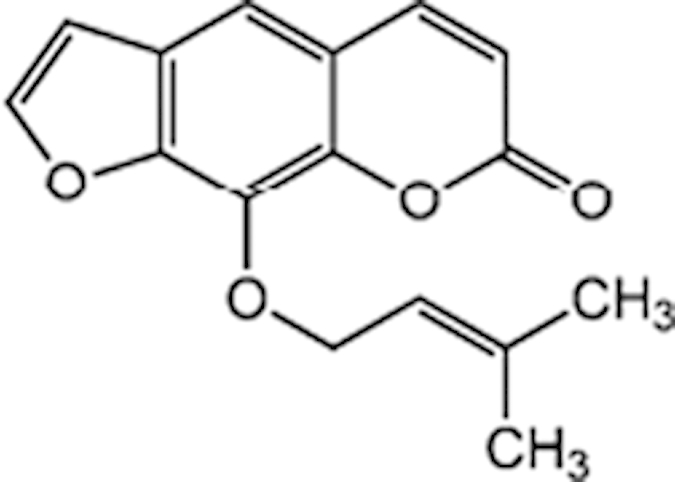	10.7 (146%)	14 (238%)	Cyp2b10 inducer
Flavone (525-82-6)	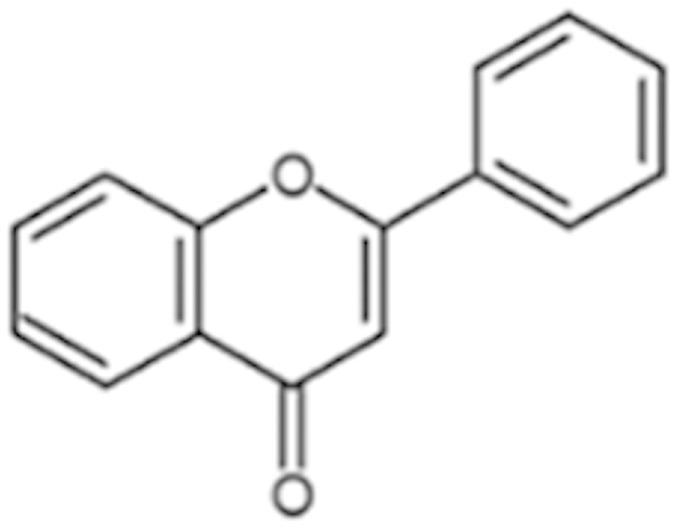	34 (184%)	32.4 (200%)	Dietary flavonoids activate CAR
Artemotil (75887-54-6)	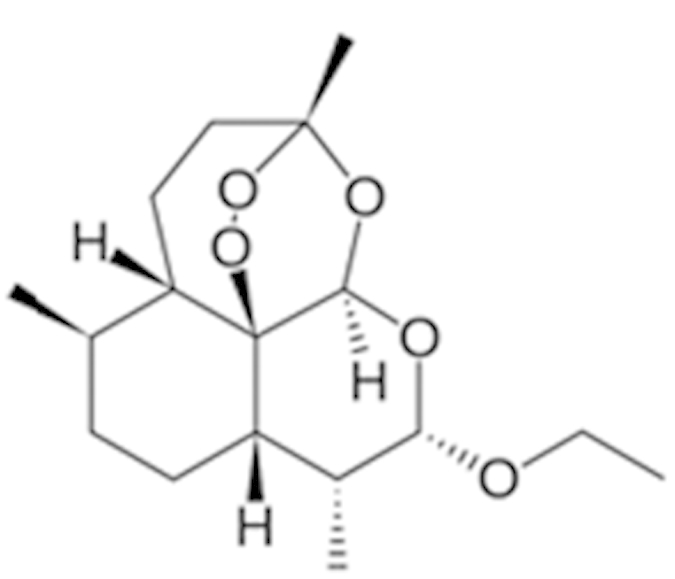	2.1 (33%)	5 (71%)	Activates CAR and Induces CYP2B6
Clebopride (55905-53-8)	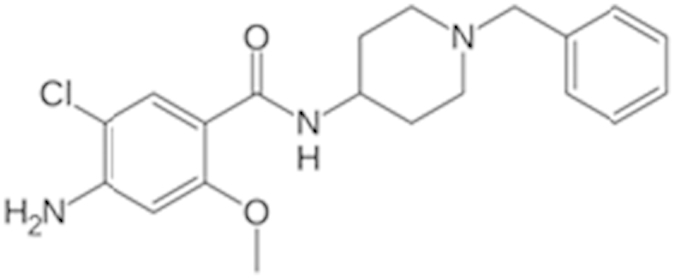	7.6 (52%)	9.4 (80%)	

**Table 2:** Antagonist potency (IC_50_), efficacy (% in parenthesis), and cell viability in qHTS.

**Compound Name (CAS No.)**	**Chemical Structure**	**Primary Screen Potency [μM] (Efficacy)**	**Follow-up Confirmation Potency [μM] (Efficacy)**	Cytotoxicity* **Potency [μM] (Efficacy)**	**Known modulator of CAR, CYP2B6, or CYP3A4?**
Trifluridine (70-00-8)	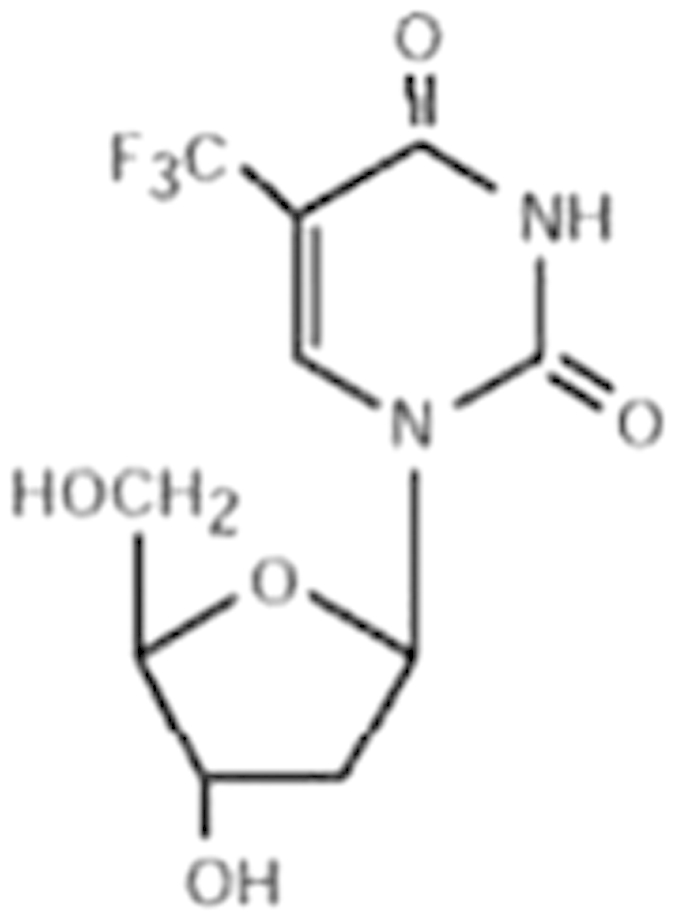	6.7 (-51%)	4.5 (-73%)	Inactive	
Bosutinib (380843-75-4)	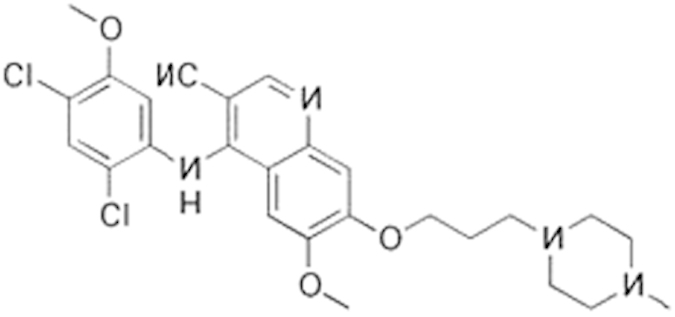	0.67 (-61%)	13.32 (-135%)	Inactive	
Ouabain (11018-89-6)	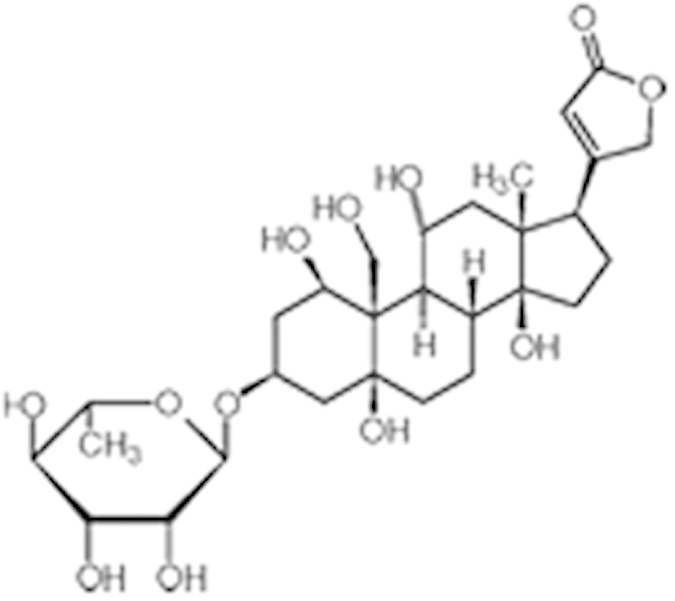	0.30 (-104%)	0.47 (-99%)	Inactive	
Digitoxin (71-63-6)	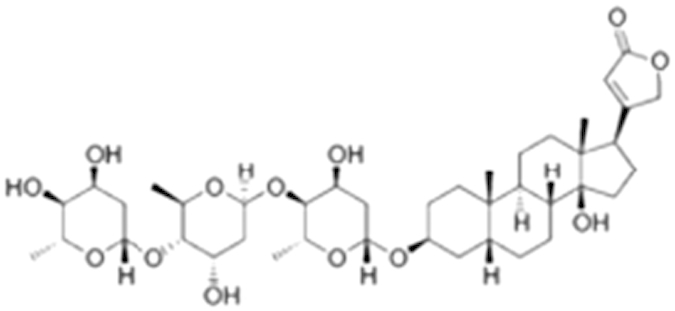	0.08 (-76%)	0.29 (-98%)	Inactive	
Nelfinavir (159989-64-7)	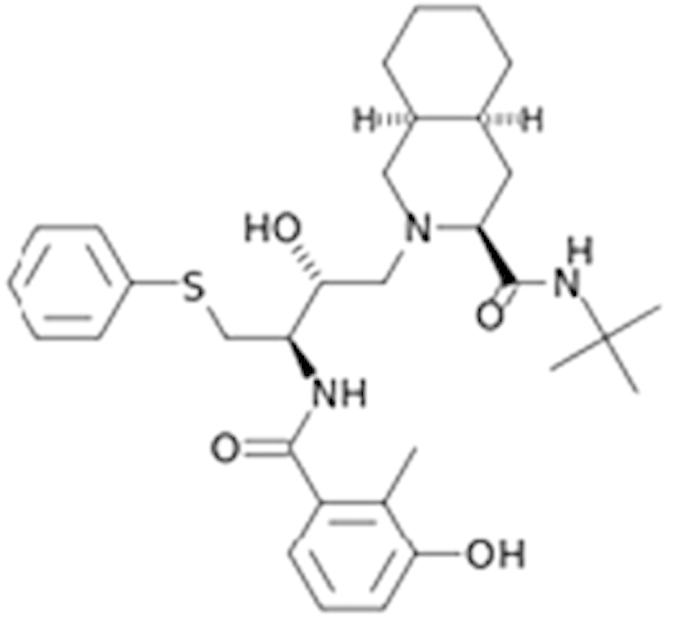	9.52 (-88%)	11 (-101%)	Inactive	
Adefovir (142340-99-6)	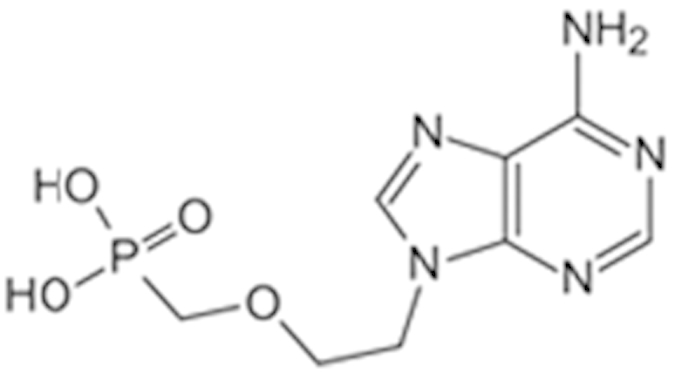	0.76 (-102%)	0.77 (-87%)	Inactive	CYP3A4 inhibitor
Mitomycin C (50-07-7)	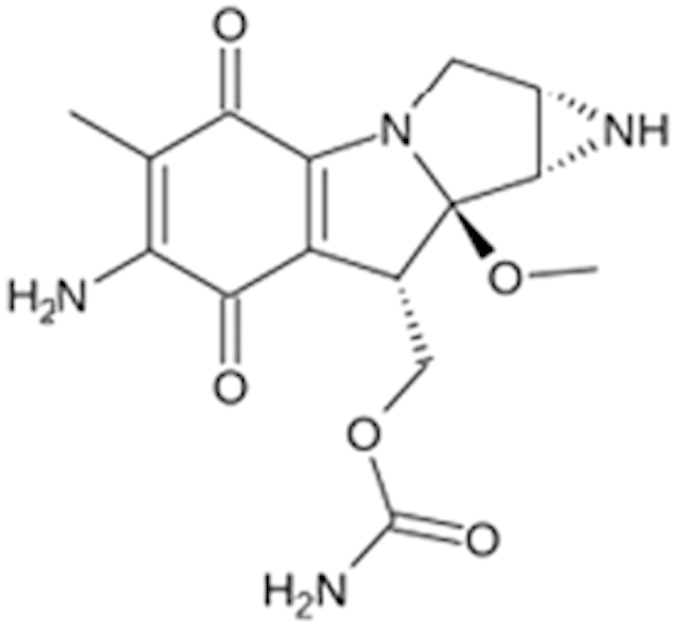	0.43 (-91%)	0.75 (-110%)	Inactive	PXR deactivator
Topotecan (119413-54-6)	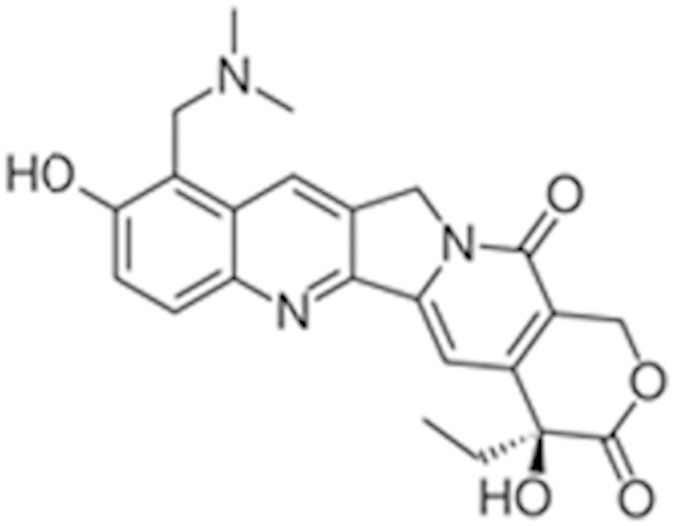	0.06 (-94%)	0.36 (-109%)	Inactive	
Daunorubicin (23541-50-6)	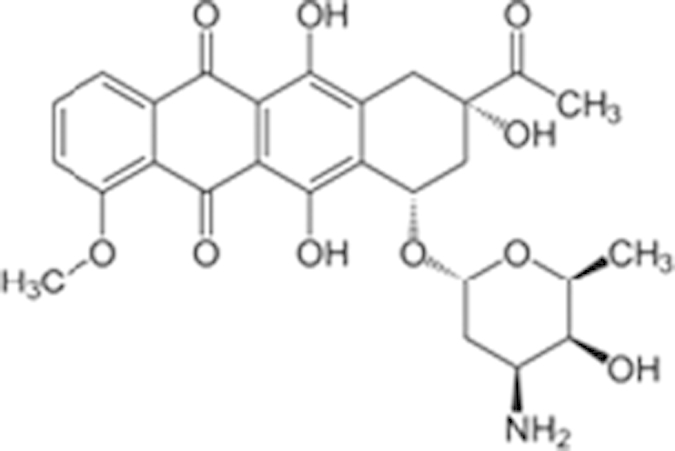	0.60 (-87%)	0.19 (-104%)	15.9 (-43%)	
Bortezomib (179324-69-7)	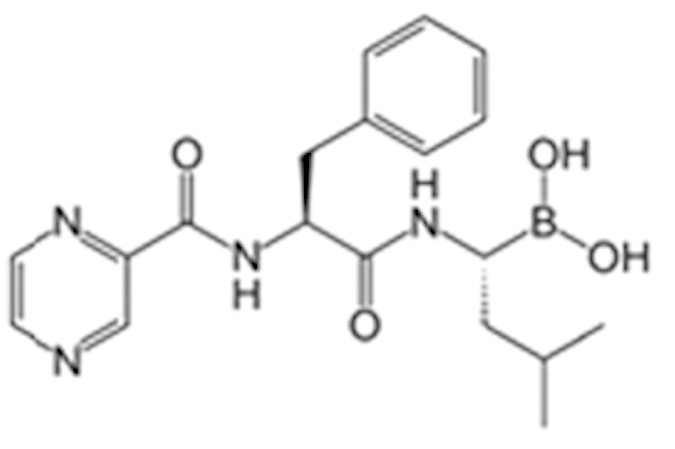	0.02 (-103%)	0.03 (-101%)	1.25 (-57%)	

^*^considered inactive if efficacy is less than 30%.
