# Supplementary material for: Quantitative High-Throughput Identification of Drugs as Modulators of Human Constitutive Androstane Receptor
Source: Sci Rep. 2015 May 20;5:10405. doi: 10.1038/srep10405 (PMC4438668; doi:10.1038/srep10405)
Supplement: Supplementary Information [file srep10405-s1.pdf]

Supplementary data to:

Quantitative High-Throughput Identification of Drugs as Modulators of Human Constitutive Androstane Receptor

Caitlin Lynch[1], Jinghua Zhao[2], Ruili Huang[2], Jingwei Xiao[1], Linhao Li[1], Scott Heyward[3], Menghang Xia[2], and Hongbing Wang[1]*

1: Department of Pharmaceutical Sciences, University of Maryland School of Pharmacy, Baltimore, Maryland 21201; 2: National Center for Advancing Translational Sciences, National Institutes of Health, Bethesda, Maryland 20892; 3: Bioreclamation IVT, Baltimore, Maryland 20

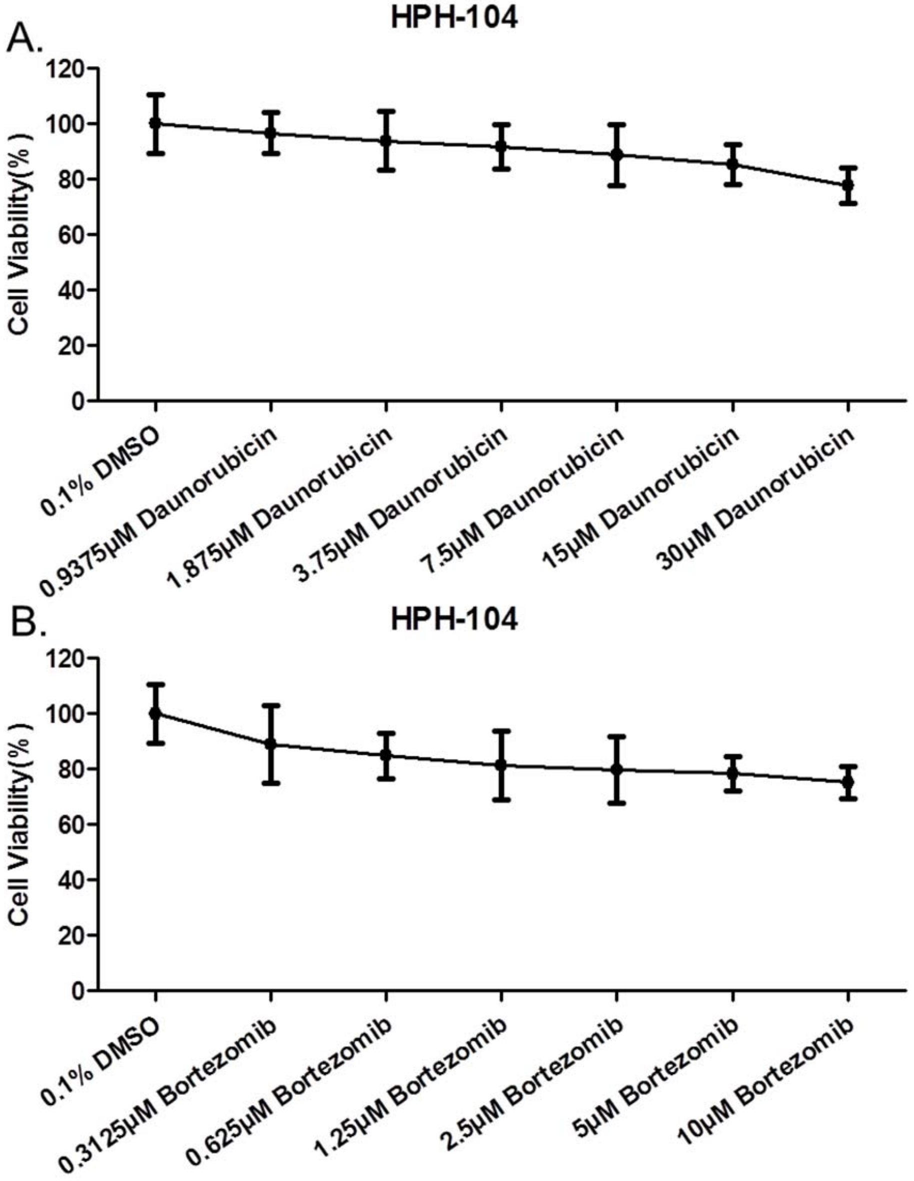

Figure S1. Cytotoxicity of daunorubicin and bortezomib in HPH. Human primary hepatocytes (from donor HL-104) cultured in 96-well collagen coated plates were treated with 0.1% DMSO, daunorubicin, or bortezomib at indicated concentrations for 24 hours. MTT assay was carried out following the manufacturer’s instruction. Data represent the mean ± SD (n =3).

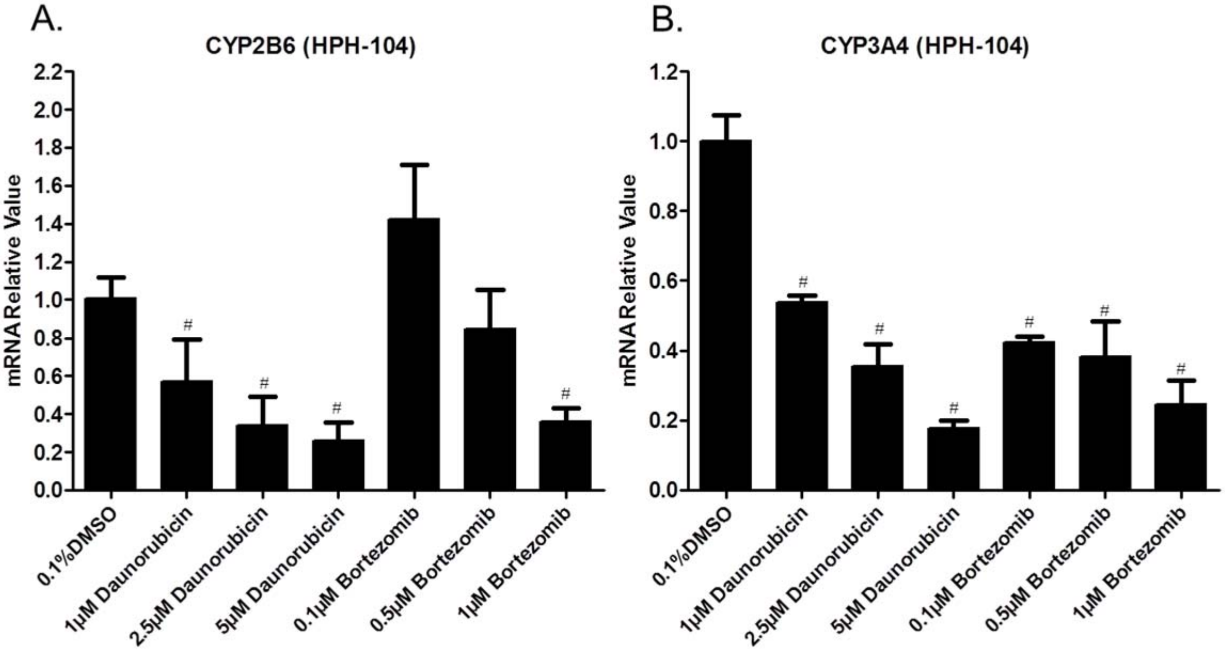

Figure S2. Effects of daunorubicin and bortezomib on the expression of CYP2B6 and CYP3A4 in HPH. HPH (from donor HL-104) were treated with the vehicle control (0.1% DMSO), daunorubicin (1, 2.5 and 5 µM), or bortezomib (0.1, 0.5, and 1 µM) for 24 h. Real-time PCR was used to analyze the expression of CYP2B6 (A) and CYP3A4 (B). Data represents the mean ± SD (n=3). #, $P < 0.05$.
